# RGDiNet: Efficient Onboard Object Detection with Faster R-CNN for Air-to-Ground Surveillance

**DOI:** 10.3390/s21051677

**Published:** 2021-03-01

**Authors:** Jongwon Kim, Jeongho Cho

**Affiliations:** Department of Electrical Engineering, Soonchunhyang University, Asan 31538, Korea; jw.kim@sch.ac.kr

**Keywords:** onboard detection, UAV, faster R-CNN, air-to-ground surveillance

## Abstract

An essential component for the autonomous flight or air-to-ground surveillance of a UAV is an object detection device. It must possess a high detection accuracy and requires real-time data processing to be employed for various tasks such as search and rescue, object tracking and disaster analysis. With the recent advancements in multimodal data-based object detection architectures, autonomous driving technology has significantly improved, and the latest algorithm has achieved an average precision of up to 96%. However, these remarkable advances may be unsuitable for the image processing of UAV aerial data directly onboard for object detection because of the following major problems: (1) Objects in aerial views generally have a smaller size than in an image and they are uneven and sparsely distributed throughout an image; (2) Objects are exposed to various environmental changes, such as occlusion and background interference; and (3) The payload weight of a UAV is limited. Thus, we propose employing a new real-time onboard object detection architecture, an RGB aerial image and a point cloud data (PCD) depth map image network (RGDiNet). A faster region-based convolutional neural network was used as the baseline detection network and an RGD, an integration of the RGB aerial image and the depth map reconstructed by the light detection and ranging PCD, was utilized as an input for computational efficiency. Performance tests and evaluation of the proposed RGDiNet were conducted under various operating conditions using hand-labeled aerial datasets. Consequently, it was shown that the proposed method has a superior performance for the detection of vehicles and pedestrians than conventional vision-based methods.

## 1. Introduction

Unmanned aerial vehicles (UAVs), also known as drones, have been developed primarily for military purposes and are used in universities and research institutions for research in computer science and robotics. Studies focus mainly on autonomous flight-based search and rescue [1], shipping [2], agriculture [3] and infrastructure inspection [4]. One of the most important components for the autonomous flights or air-to-ground surveillance of UAVs is object detection, which requires real-time data processing with high accuracy. It is directly utilized in diverse tasks such as situation analysis, traffic monitoring, disaster analysis and object tracking. Particularly, when flying in cities or residential areas, it is extremely important to detect and avoid objects because of safety issues arising from the terrain, obstacles, collisions with other objects, and falls [5].

Object detection is a fundamental topic in computer vision and an important component of sensor-based situational awareness systems for autonomous driving. With the emergence of convolutional neural networks (CNNs), object detection schemes have evolved significantly in the past few years. The development of various detection frameworks, such as faster region-based CNN (R-CNN) [6], RetinaNet [7], and single-shot multi-box detector (SSD) [8], has led to considerable advancement in the newly invented state-of-the-art technologies for object detection and identification. Two-phase detection frameworks including preprocessing steps to generate region proposals, such as faster R-CNN and mask R-CNN [9,10], have been proposed to increase the detection accuracy. Moreover, single-phase detection frameworks, such as “you only look once” (YOLO) [11], Single Shot Detector (SSD), and corner-Net [12], have been developed to maximize the computational efficiency to enable real-time detection. Despite the clear differences between these two pipeline structures, the ultimate objectives of the modern detectors being currently proposed are to accurately classify objects and estimate their locations in images. Although, concurrently, numerous different object detection frameworks have been proposed [13,14,15], designing a robust and accurate detection system is still a challenging task because of the various environmental problems encountered in real scenarios.

Monocular RGB cameras are being utilized to develop numerous object detection techniques, and they are the most common sensors and primary options for detecting various objects, including traffic signs, license plates, pedestrians and cars. However, passive vision systems, such as cameras, cannot be used for night vision, cannot acquire information about depth, and are limited in use in real driving scenarios because of their sensitivity to the changes in brightness [16,17]. To resolve the drawbacks of such cameras, employing object detection techniques using multimodal data, instead of using cameras only, has recently been proposed. Several methods have proposed utilizing the depth information collected from stereo vision or infrared (IR) sensors and integrating them with RGB images [18,19]. They have achieved significant performance improvements compared to monocular camera-only-based methods. Furthermore, regarding autonomous driving, three-dimensional (3D) sensors, such as light detection and ranging (LiDAR) sensors, are more common than 2D vision sensors because safety requires higher accuracy [20]. Most conventional techniques first utilize LiDAR to convert point cloud data (PCD) into images by projection [21,22] or into a volumetric grid via quantization, and subsequently apply a convolutional network [23,24]. Recently, it has been proposed to directly deal with point clouds without converting them into other formats [25].

Consequently, the recent advances in multimodal data-based object detection have made significant progress in autonomous driving technology, and the KITTI benchmark [26] site reports that state-of-the-art algorithms can achieve an average precision (AP) of up to 96%. Although such remarkable advances have already been achieved in object detection, they may be unsuitable for processing the sequences or images captured by UAVs. This may be attributed to the following major problems: (1) objects generally have a smaller scale than images; (2) objects are typically nonuniformly and sparsely distributed throughout an image; (3) objects experience various environmental changes, for example, occlusion and background interference; (4) the payload weight of a UAV is limited. The features of object detection in air-to-ground views and on-road scenarios are compared in Table 1.

In aerial images, the visual distance from a UAV to an object can be long and constantly changing, so that the object of interest appears small and contains less information about its features. Simultaneously, the field of view of the target scene is wide and the object to be detected experiences various environmental changes, such as background interference, shadows of other objects and occlusions. Moreover, owing to the payload weight limitations, it is difficult to mount high-performance processors simultaneously with multiple sensors. However, even if they could be mounted, there are few available techniques for locating objects by multimodal data integration and it is also difficult to ensure robustness against noise. For example, in [27], a simple CNN-based approach for automatic object detection in aerial images was presented, and [28,29,30] directly extended a faster R-CNN to object detection in aerial images. Similarly, the detection algorithms proposed in [31,32,33] were based on a single-stage framework, such as SSD and YOLO. Recently, to resolve the ambiguities of viewpoints and visual distances, [34] proposed a scale adaptive proposal network and [35] suggested utilizing reinforcement learning to sequentially select the areas to be detected on a high-resolution scale. These methods aim to efficiently detect vehicles or pedestrians on roads and accurately count the number of objects, and can be employed for the detection of objects in aerial images. Because object detection based on monocular RGB cameras still has significant limitations, as addressed earlier, some authors have proposed new techniques using thermal imaging sensors [36,37] and depth maps of infrared (IR) sensors [38,39]. However, they also have a limitation in that the distance to an object is unknown; thus, they can only locate an object indoors or within 10 m. Consequently, it is difficult to obtain satisfactory detection results in aerial views using the existing technologies and the detection schemes, if any, proposed in the literature focus only on vision sensor-based object detection in aerial images. The abovementioned issues motivated us to devise a framework for efficiently detecting objects in air-to-ground views while measuring the distance from a UAV to the target.

**Figure 1:**
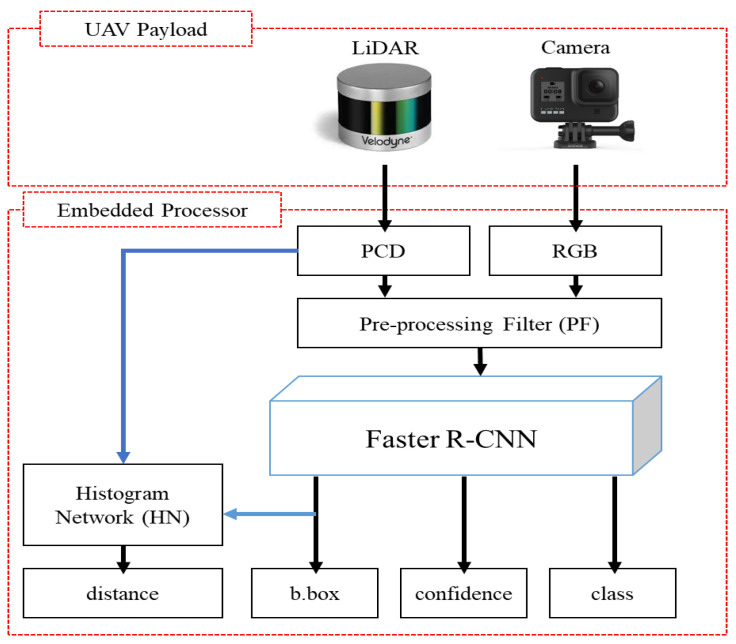
Architecture of proposed multimodal aerial data-based object detection system.

In this paper, we propose a new real-time object detection system based on RGB aerial images and a PCD depth map image network (RGDiNet), which can provide improved object detection performance as well as the distance to objects in aviation. Moreover, we verify its performance using a UAV embedded with an Nvidia AGX Xavier prototype. The results show that the proposed system can perform powerful real-time onboard detection in aviation for scenes with varying image conditions and various environments. Figure 1 displays the proposed detection system, which comprises a preprocessing filter (PF) for generating a newly integrated color image, a faster R-CNN as the baseline detection network, and a histogram network (HN) for predicting the distance from the UAV to objects. Based on the aerial observations (i.e., RGB images and PCD) captured by the UAV, the PF produces a three-channel fused image consisting of a portion of the RGB channels and a depth map created by the point clouds. The reconstructed image is applied to the faster R-CNN to predict the bounding box, class and confidence score of each object. Subsequently, the distance is calculated using the HN with the predicted bounding box and the PCD.

Compared with the conventional detection methods based on multimodal data, the proposed RGDiNet has several advantages: (1) Utilizing the PF, the learning framework does not require a connection layer, 1 × 1 convolutional layer, or sub-network to integrate two data points. Therefore, our approach can significantly reduce the computational costs and improve the detection efficiency; (2) Using the HN, the actual distance between a predicted object and a UAV can be provided; and (3) The 3D PCD can be used with simultaneous localization and mapping (SLAM) [40] for object positioning along with the surrounding environment, if necessary, and it also has robustness against changes in the imaging conditions and various environments. Detection frameworks combining two different inputs have been proposed [41]; however, contrastingly, we propose a method to substitute one color channel in an RGB image with another signal. This approach is assumed to be efficient in the onboard systems of UAVs owing to the simplification of the network structure. After analyzing the object detection impact diagram for the R, G, and B channels, the less affected channel is substituted by a depth map created by the PCD to reconstruct the input for the faster R-CNN.

The proposed object detection algorithm for autonomous flights or ground surveillance was tested in various operating environments. Hand-labeled aerial datasets containing two categories of objects (pedestrians and vehicles) were used for the evaluation of its performance. The experimental results showed that the proposed system improved the object detection performance by increasing the AP by almost 5% even in a noisy environment or poor conditions in which the brightness varied. Such improved performance is expected to be practical by expanding the proposed object detection scheme to various fields, such as autonomous flying drones and ground surveillance from an air-to-ground view.

## 2. Methodology

### 2.1. System Overview

The proposed multimodal data-based detection architecture, RGDiNet, can detect objects in aerial images in which the shapes and dynamic environment of the objects are constantly changing. A LiDAR and a camera are fixed on a UAV, and it is assumed that both the sensors consider only objects present within 40 m. Moreover, the sensors are synchronized. The pipeline of RGDiNet consists of three tasks: the generation of PCD depth maps and RGB images by the PF, object detection and bounding box prediction by the faster R-CNN, and calculation of the distance between the UAV and an object by the HN. The detection is based on the faster R-CNN utilizing a region proposal network (RPN), and the newly integrated image reconstructed by the PF is input into the faster R-CNN to predict the bounding box containing the entity of interest and its confidence score. Subsequently, the coordinates of the bounding box are projected onto the PCD to calculate the actual position of the detected object. Finally, the PCD within the bounding box are quantified to compute the average distance to this object.

### 2.2. Integration of RGB Image and PCD Depth Map

To use the data obtained from sensors with mismatching dimensions, such as a camera and a LiDAR, preprocessing should be conducted. An RGB image can be used directly from a camera without any processing. However, a LiDAR returns readings in spherical coordinates; therefore, the position of a point in 3D space needs to be converted into a position on the surface of the image in pixel coordinates. To build a two-dimensional (2D) depth map, first, the 3D PCD are transformed into a front view using the rotation transformation expressed in Equation (1) to match the 2D RGB image.

(1) $$\left[\begin{array}{c} x^{\prime }\\ y^{\prime }\\ z^{\prime } \end{array}\right]=\left[\begin{array}{ccc} \cos\left(\theta_{R}\right) & 0 & -\sin\left(\theta_{R}\right)\\ 0 & 1 & 0\\ \sin\left(\theta_{R}\right) & 0 & \cos\left(\theta_{R}\right) \end{array}\right]\;\;\;\;\;\;\;\left[\begin{array}{c} x\\ y\\ z \end{array}\right].$$

**Figure 2:**
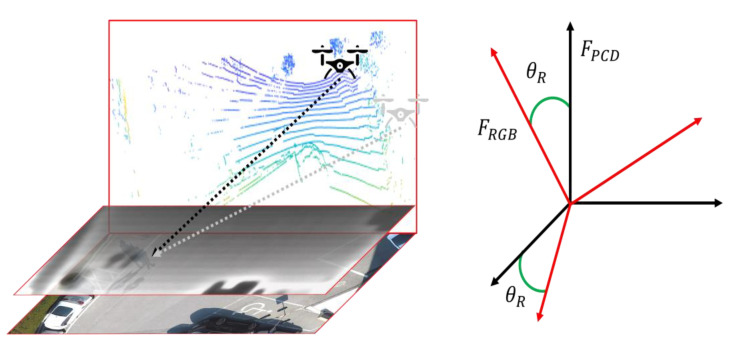
Projection of point clouds in 3D space on 2D surface.

Here, ${\left[x,y,z\right]}^{T}$ is the 3D vector of the PCD and, using Equation (1), the vector is rotated by an angle $\theta_{R}$ about the y-axis. Considering a drone flying over the surface of an image plane, as shown in Figure 2, the PCD are created based on the absolute coordinates, $F_{PCD}$. To project them on the image in pixel coordinates, the absolute coordinates, $F_{PCD}$, are rotated by the angle, $\theta_{R}$, to convert into $F_{RGB}$. In this study, the PCD were collected in the direction normal to the ground at a fixed angle of 60° about the y-axis. 

Assuming that the LiDAR is calibrated by the image plane of the camera, the projection of the points of the LiDAR appears much sparser than that in its associated RGB image. Because the proposed object detection system employs a 16-channel LiDAR, instead of the 64-channel one commonly used on the ground, the scarcity of the data distribution is inevitably worse. Because such limited spatial resolution obtained from a LiDAR sensor makes object detection very challenging, we adopted the bidirectional filter-based strategy proposed in [42] to reconstruct the PCD into a high-resolution depth map. This filtering technique has the merits of high computational efficiency and easy parameter adjustment.

Once the depth map is built, it is concatenated with the RGB image to create a reconstructed input space. RGB, which represents red, green, and blue, is the primary color space of a computer for capturing or displaying images. The human eye is sensitive to these three primary colors [43]. Specifically, we can easily recognize and detect objects of more sensitive wavelengths, such as ambulances and warning signs. Based on this background, we assumed that certain color channels have little influence on object detection. The effect of the selection of the color space, such as RGB, CMY, and YCrCb, on the detectability and classification of objects was studied in [44]; however, no studies have been conducted on the influence of specific channels on object detection performance. Therefore, we trained the object detection algorithm by removing channels from the RGB image sequentially, and analyzed the effect of the color of each channel on the object detection. After this analysis, the channel that was least affected was replaced by a depth map, and the reconstructed image was adopted for the object detection. A simple experiment confirmed that the blue channel lowered the object detection performance by up to 3%, similar to human vision; therefore, the blue channel was replaced by a depth map. Thus, the proposed object detection network in the UAV, using a depth map instead of the blue channel of RGB, was named RGDiNet. It is the most direct extension of a faster R-CNN through multiple modalities because only a part of the input image is replaced by a depth map. Once the faster R-CNN is trained and the reconstructed input image is ready by extracting the depth map by the PF, all the object detection preparations are completed.

### 2.3. Object Detection in Aerial Image Using Faster R-CNN

**Figure 3:**
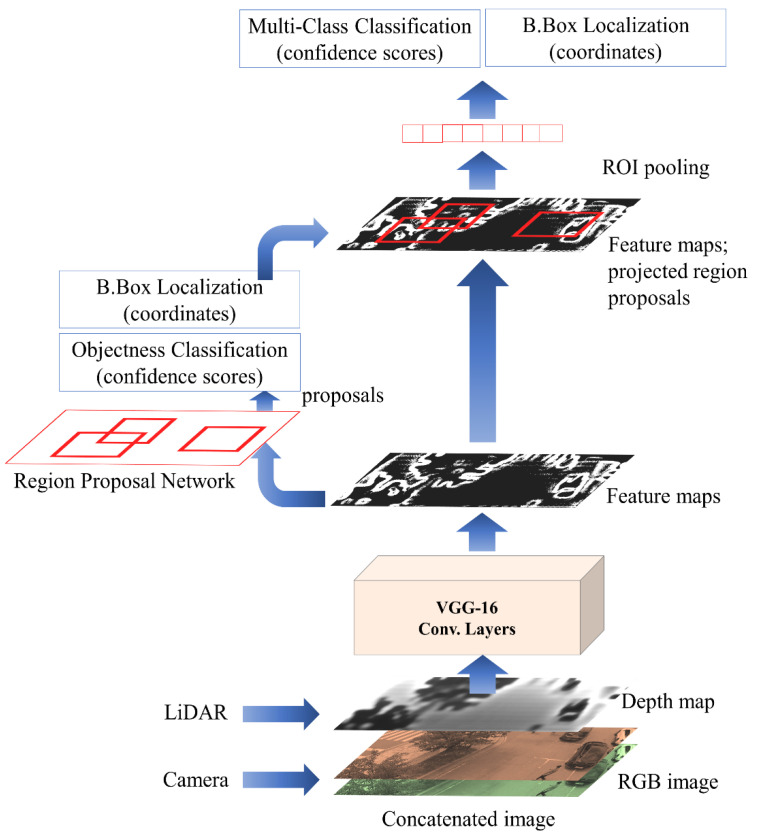
Faster region-based convolutional neural network (R-CNN) pipeline for object detection by integration of a camera and light detection and ranging (LiDAR).

As described earlier, the proposed RGDiNet utilizes a faster R-CNN to detect objects based on a reconstructed RGD image. Figure 3 shows the object detection pipeline using the faster R-CNN. As a backbone architecture for learning image features, a network is built based on VGG-16 [45] and is initialized with pretrained weights using the ImageNet dataset [46]. The core concept of the faster R-CNN is an RPN. While inheriting the existing fast R-CNN structure, the selective search is removed and the region of interest (ROI) is calculated by the RPN. Overall, a feature map is first extracted by the CNN and subsequently sent to the RPN to calculate the region of interest (ROI). The RPN is a fully convolutional network, which effectively generates a wide range of region proposals that are delivered to the detection network. Region proposals are rectangular in shape and may or may not include candidate entities. A detection network is connected after the RPN to refine the proposals, and several ROIs are passed as inputs. A fixed-length feature vector is extracted for each ROI by an ROI pooling layer. The network creates region proposals by sliding a small window on the shared convolutional feature map. In the last convolutional layer, 3 × 3 windows are selected to reduce the dimensionality of the representation, and the features are provided in two 1 × 1 convolutional layers. One of them is for localization and the other is to categorize the box as the background or the foreground [47]. Herein, the faster R-CNN provides the probabilities for the two classes of vehicles and pedestrians viewed from the UAV as well as the coordinates of their bounding boxes. The RPN generates a candidate list of bounding boxes from the RGD aerial image, each associated with a confidence score that measures the memberships of selected parts of an image against a set of object classes versus the background.

### 2.4. Estimation of Visual Distance Using Histogram Network

The 3D PCD from the LiDAR are projected onto the 2D image plane to obtain the visual distance to the target. However, it is impossible to project directly onto the image plane because the PCD are essentially created in the spatial coordinate system of 3D point clouds. Thus, the coordinate points of the bounding box predicted based on the image are projected onto the PCD plane using data voxelization [48,49,50], a classical method commonly used for quantifying point clouds.

**Figure 4:**
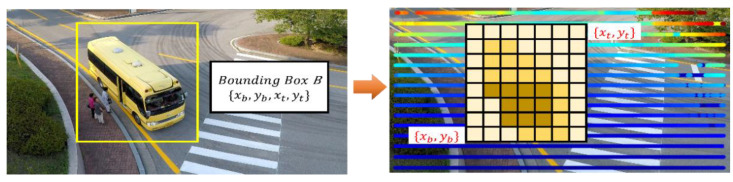
Schematic of estimation of visual distance to target.

In the proposed RGDiNet, the PCD use a two-point-five-dimensional self-centered occupancy grid map, in which each voxel covers a small ground patch of 0.1 m × 0.1 m. Each cell stores a single value indicating how occupation of the voxel by the object or background. Figure 4 shows a schematic of the estimation of the visual distance to the target. After voxelization, the voxelized PCD are normalized to match the projected points of the bounding box, $\left\{x_{b},y_{b},x_{t},y_{t}\right\}$, and the image coordinate system to the PCD plane. $\left\{x_{b},y_{b}\right\}$ and $\left\{x_{t},y_{t}\right\}$ are the coordinates of the bottom left and top right sides of the bounding box, respectively. Subsequently, the average of the occupation values of the voxels in the projected bounding box is considered as the distance to the target.

## 3. Experimental Results

### 3.1. Assessment Preliminaries

The major benefit of the proposed RGDiNet is that it can detect on-road objects and measure distances from an air-to-ground view by integrating the images from heterogeneous sensors. This enables the accurate detection of objects in various aviation environments, ultimately facilitating ground surveillance and safe autonomous flights. The proposed algorithm was comprehensively evaluated using a hand-labeled dataset, called the SCHF dataset, to verify its use by mounting on an actual autonomous flight system. The SCHF dataset consists of 2428 densely labeled (i.e., pedestrian and vehicle) pairs of aligned RGB and RGD multimodal images. Once the faster R-CNN model was trained on an offline workstation, the optimized model was installed on an aerial platform to evaluate the real-time detection performance. Assuming a virtual flight, the test dataset was used to validate the performance of the proposed system. The airborne platform used in this study was a DJI Matrice 600 Pro quadcopter integrated with a stabilized gimbal. Note that to stabilize the training process of the faster R-CNN, we adopted the standard loss function with the same hyperparameter as in [48].

**Figure 5:**
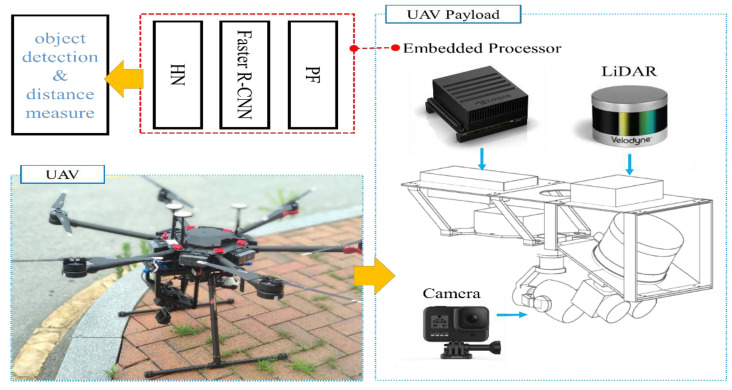
Unmanned Aerial Vehicle (UAV) designed for real-time data collection, processing, and test evaluation using RGDiNet.

RGB images were acquired by a Gopro Hero 2019 edition camera mounted on a UAV, having a video resolution of 1920 × 1080 and a frame rate of 30 frames per second (FPS). In addition, the PCD were collected by a VLP-16 LiDAR mounted on the UAV; the LiDAR had 16 channels and a frame rate of 10 FPS. A high-performance embedded system module, NVIDIA JETSON Xavier, was installed onboard the UAV for air flight condition monitoring as well as signal processing of the PF, detectors, and HN comprising the proposed RGDiNet. The prototype UAV bus and payload based on NVIDIA JETSON Xavier is shown in Figure 5.

**Figure 6:**
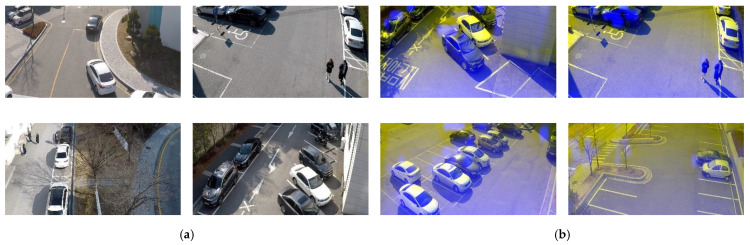
Example images from SCHF dataset (**a**) RGB image (**b**) RGD image.

The SCHF dataset was prepared to train the proposed RGDiNet and conduct its performance evaluation. The following three main aspects were ensured when collecting the data: (1) fixed sensor angle of 60°; (2) collection of images of objects of various scales and aspect ratios from 15 to 30 m away; and (3) fixed resolution as 1700 × 700 by image calibration. The video shot on the UAV covered ten different parking lots and two-lane roads. For each location, a 10-min-long video was recorded and “PNG” image files were generated at 1-s intervals. The SCHF dataset contains 3428 frames with 5020 object labels for two categories: pedestrians (1638) and vehicles (3382). In addition, two types of RGB images and multimodal images (RGD) were created for comparison with the results of an object detection system utilizing only RGB images. The dataset was split into two sets: 75% for training and 25% for testing. The details of the dataset are summarized in Table 2, and example images are shown in Figure 6.

### 3.2. Performance Evaluation

**Figure 7:**
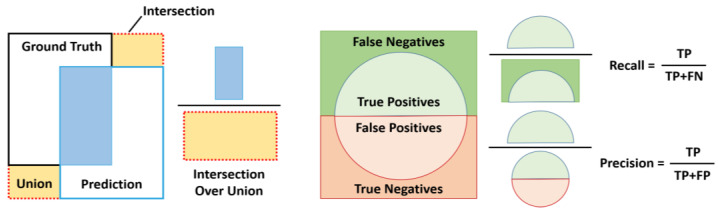
Illustrations of intersection over union (IoU), precision and recall.

Generally, object detection performance is evaluated to verify the presence of an object image as well as the ability to determine the accurate location of the object in the image [51,52]. Three common indicators were used to assess the detection performance of the proposed RGDiNet: intersection over union (IoU), precision-recall curve (PRC), and AP. For discrete scores, each detection is considered positive if the ratio of the IoU to a one-to-one matched ground-truth annotation is greater than 0.5 or 0.7. By changing the detection score threshold, a set of true positives, true negatives, false positives, and false negatives is created, based on which the PRC and the AP are calculated. Figure 7 depicts the definitions of IoU, precision, and recall. 

Two types of comparative experiments were conducted to evaluate the performance of the proposed system. Specifically, RGDiNet was compared to a general faster R-CNN model that only uses RGB images under normal conditions and in a noisy environment. The evaluation results are summarized in Table 3. The IoU was fixed at 0.5 and the APs of the two models were compared for two objects (vehicle and pedestrian). RGDiNet showed an AP at least 1.5% points higher than the vision-based faster R-CNN, confirming its superiority. By including the PF and the HN, the processing time of RGDiNet was measured 0.1 s slower than that of the vision-based faster R-CNN. However, this is not considered a long time to be critical for data processing, and thus, real-time operation is possible.

**Figure 8:**
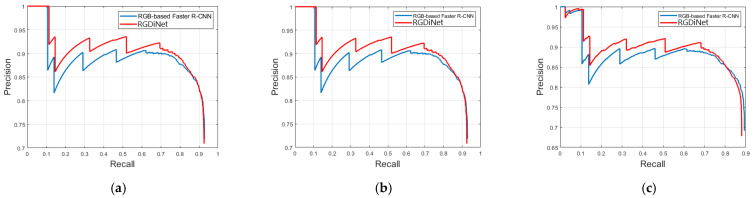
AP comparison of vision-based faster R-CNN and RGDiNet with varying IoUs: (**a**) IoU = 0.3 (**b**) IoU = 0.5 (**c**) IoU = 0.7.

In addition, more detailed comparisons were made using the PRC. Figure 8 compares the PRCs of the conventional vision-based faster R-CNN and RGDiNet with varying IoUs. In the figure, the following are observed: (1) RGDiNet surpasses the existing vision-based faster R-CNN model for all the IoU thresholds. Specifically, at a high IoU, such as 0.7, RGDiNet can still locate objects very accurately without using other networks; (2) The precisions of RGDiNet and the existing model are similar at very high recall rates; and (3) Because RGDiNet can measure the visual distance using the PCD, it has a unique advantage of providing the information of the visual distance to real objects.

**Figure 9:**
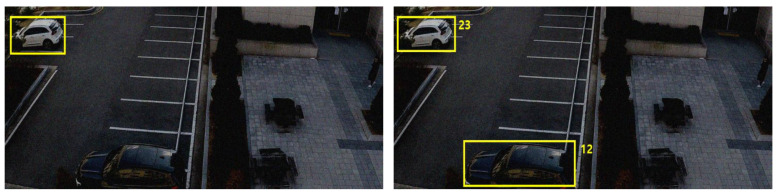

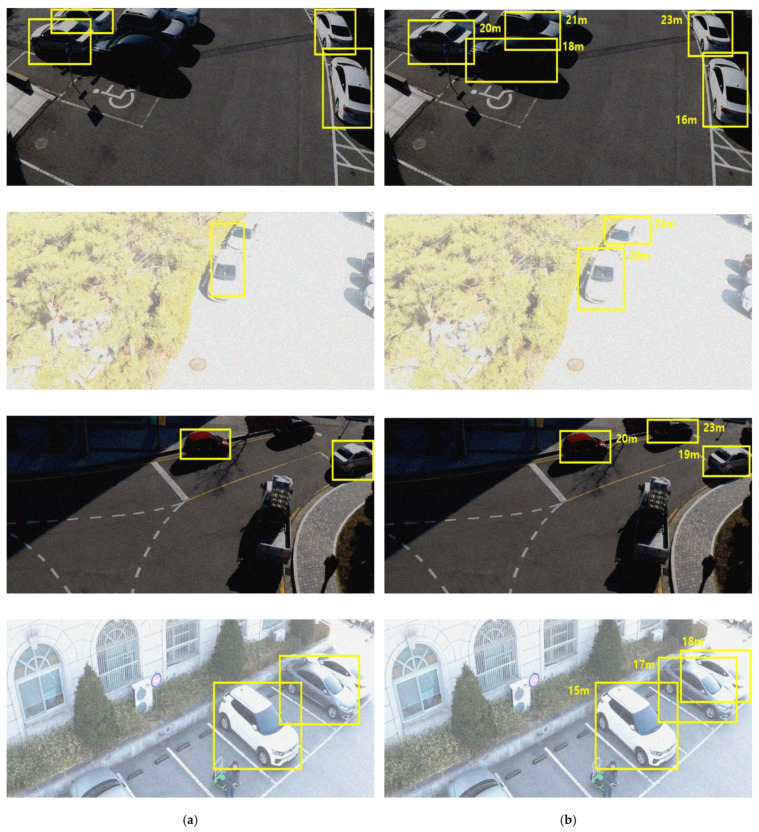
Comparison of vehicle detection results by vision-based faster R-CNN (**a**) and RGDiNet (**b**).

**Figure 10:**
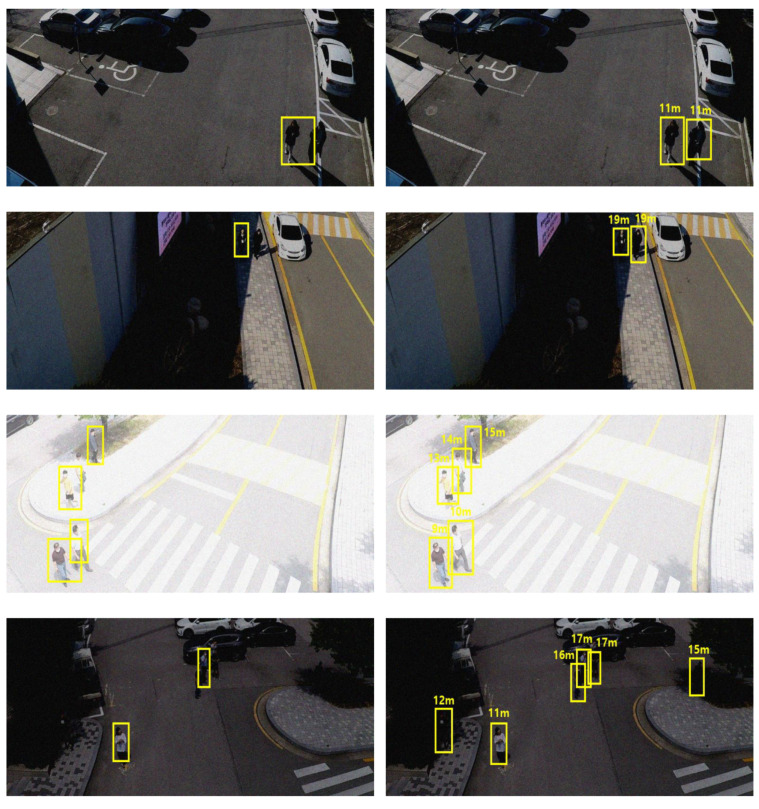

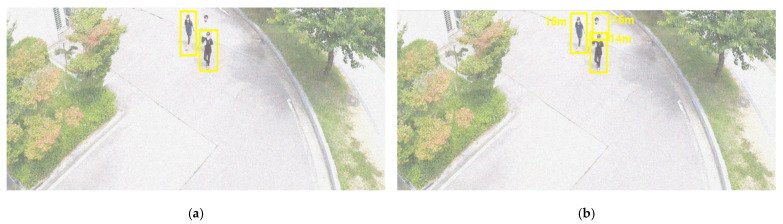
Comparison of pedestrian detection results by vision-based faster R-CNN (**a**) and RGDiNet (**b**).

LiDAR has a high level of robustness against environmental changes. To confirm its intrinsic robustness even in the proposed simple input convergence network structure, additional experiments were conducted in noise. Gaussian and Poisson noises, with zero mean and unit variance scaled by 0.1, which may occur during video and electronic signal processing, were considered and their effects on the performance were examined. We also tested the robustness of the proposed system against light noise by adding randomly generated values for all the pixels in the original image, considering the changes in the ambient brightness due to weather conditions. The change in the brightness was classified into two categories: change from −75 to 75 (weak) and change from −125 to 125 (strong). The results are summarized in Table 4. The image distortion caused by the changes in the ambient brightness degraded the overall performance of all the detectors compared to that under normal conditions. However, the proposed RGDiNet demonstrated a more robust noise tolerance than the vision-based faster R-CNN. A higher robustness was found particularly when the Gaussian noise was delivered. Figure 9 and Figure 10 show the detection results for each class in a noisy environment. It is confirmed, as before, that the existing vision-based detection method is sensitive to the environmental changes of increased darkness or brightness, and thus, there were numerous cases of failure in the detection of vehicles or pedestrians.

Summarizing, the proposed RGDiNet demonstrated its superiority by showing 1–2% higher AP at all the thresholds of the IoU than the conventional vision-based object detection method based on the performance test results using hand-labeled aerial data. The processing speed per image was less than 1 s on average, ensuring the possibility of real-time application. In addition, the proposed RGDiNet maintained an AP of 5% higher than the existing vision-based model and confirmed its superiority in the performance evaluation performed on noise addition noise to verify the robustness against environmental changes.

## 4. Conclusions

Recent advances in multimodal data-based object detection frameworks have led to significant improvements in autonomous driving technologies and the KITTI benchmark site reports that the latest algorithm can achieve an AP of up to 96%. However, these remarkable advances may be unsuitable for image processing for direct object detection in aerial views. It is difficult to obtain satisfactory detection results from an air-to-ground view using the existing technologies and detecting objects in aerial images based only on vision sensors, which are focused in most studies, has a drawback of being very sensitive to external environmental changes.

In this paper, we proposed RGDiNet, a new multimodal data-based real-time object detection framework, which can be mounted on a UAV and is insensitive to the changes in the brightness of the surrounding environment. In addition, the proposed scheme uses PCD to provide the visual distance to a target, unlike other existing aerial detection systems. The object detection algorithm embedded in the signal processor of the UAV is designed based on an RGD, a combination of the RGB aerial images and the depth maps reconstructed by the LiDAR PCD, for computational efficiency. The developed algorithm for autonomous flight or ground surveillance is installed on a prototype based on Nvidia AGX Xavier to process the sensor data. The evaluation of RGDiNet on hand-labeled aerial datasets under various operating conditions confirms that its performance for the detection of vehicles and pedestrians is superior to that of conventional vision-based methods, while processing the data at a real-time level of 1–2 FPS. Therefore, the practical applications of autonomous flying drones can be expected using the proposed detection technology. In the near future, we plan to more efficiently improve the integration process of the sensor data and improve the performance by network restructuring. These will be actually utilized and evaluated in applications, such as delivery and search-and-rescue, which are expected to be used in practice.

## Figures and Tables

**Table 1:** Comparison of features considered when detecting objects in an air-to-ground scenario.

**Features**	*On-road*	*Aviation*
**Visual distance**	*Constant*	*Varies*
**Field of view**	*Small*	*Large*
**Background complexity**	*Medium*	*High*
**Payload weight**	*Unlimited*	*Very limited*
**Exemplary images**	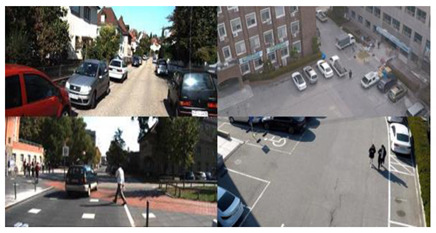	

**Table 2:** Details of SCHF dataset.

Sensor	Image Frames	Resolution	Class	
			# of Vehicles	# of Pedestrians
RGB camera + 16-CH LiDAR	3428	1700 × 700	3382	1638

**Table 3:** AP comparison of vision-based faster R-CNN and RGDiNet when IoU = 0.5 for various objects.

Method	Processing Time (s)	$AP\;\left(IoU=0.5\right)(\%)$	
		Vehicle	Pedestrian
*Vision-based faster R-CNN*	0.8	83.22	84.80
*This study (RGDiNet)*	0.9	84.78	88.33

**Table 4:** Performance comparison of object detectors in noisy environment.

Method	Class	$AP\;\left(IOU=0.5\right)(\%)$		$AP\;\left(IOU=0.5\right)(\%)$	
		$N_{gauss}\;(\mathrm{Weak})$	$N_{poiss}\;(\mathrm{Weak})$	$N_{gauss}\;(\mathrm{Strong})$	$N_{poiss}\;(\mathrm{Strong})$
*Vision-based faster R-CNN*	Vehicle	57.56	88.43	54.08	60.54
	Pedestrian	54.69	71.26	35.40	57.18
*This study (RGDiNet)*	Vehicle	62.71	89.06	58.27	60.94
	Pedestrian	59.62	73.22	36.58	57.91

## Data Availability

The data presented in this study are available on request from the corresponding author.
